# The relation between endothelial dependent flow mediated dilation of the brachial artery and coronary collateral development – a cross sectional study

**DOI:** 10.1186/1476-7120-7-25

**Published:** 2009-06-15

**Authors:** Aydan Ongun Ozdemir, Sadi Gulec, Nihal Uslu, Cansın Tulunay Kaya, Cagdas Ozdol, Sibel Turhan, Yusuf Atmaca, Timucin Altin, Cetin Erol

**Affiliations:** 1 Department of Cardiology, Ankara University School of Medicine, Ankara, Turkey; 2 Department of Radiology, Baskent University School of Medicine, Ankara, Turkey

## Abstract

**Background:**

Endothelial dysfunction is thought to be a potential mechanism for the decreased presence of coronary collaterals. The aim of the study was to investigate the association between systemic endothelial function and the extent of coronary collaterals.

**Methods:**

We investigated the association between endothelial function assessed via flow mediated dilation (FMD) of the brachial artery following reactive hyperemia and the extent of coronary collaterals graded from 0 to 3 according to Rentrop classification in a cohort of 171 consecutive patients who had high grade coronary stenosis or occlusion on their angiograms.

**Results:**

Mean age was 61 years and 75% were males. Of the 171 patients 88 (51%) had well developed collaterals (grades of 2 or 3) whereas 83 (49%) had impaired collateral development (grades of 0 or 1). Patients with poor collaterals were significantly more likely to have diabetes (*p *= 0.001), but less likely to have used statins (*p *= 0.083). FMD measurements were not significantly different among good and poor collateral groups (11.5 ± 5.6 vs. 10.4 ± 6.2% respectively, *p *= 0.214). Nitroglycerin mediated dilation was also similar (13.4 ± 5.9 vs. 12.8 ± 6.5%, *p *= 0.521).

**Conclusion:**

No significant association was found between the extent of angiographically visible coronary collaterals and systemic endothelial function assessed by FMD of the brachial artery.

## Background

Ischemic heart disease and myocardial infarction are the major causes of mortality and morbidity in industrialized countries. Well developed coronary collateral vessels have protective impacts such as limiting the size of myocardial infarction, reducing the incidence of heart failure and ventricular aneurysm, fewer cardiovascular events during follow-up and better survival rates [1-3]. However, there are significant differences in the degree of collateral development among individual patients [4]. Although clinical presentation with stable angina pectoris, presence of diabetes mellitus, the extent of coronary artery stenosis, the use of calcium channel blockers, statins, angiotensin converting enzyme inhibitors, elevated levels of C-reactive protein, and the number and function of endothelial progenitor cells were all suggested as potential determinants of collateral development, it is still unknown why some patients are capable of developing sufficient collateral circulation while others are not [5-8].

Recent studies have demonstrated that, nitric oxide (NO) bioavailability is critically important in collateral formation [9-11]. This led to a common perception that endothelial dysfunction is one of the potential mechanisms for the decreased presence of coronary collaterals. To date, however, the relation between endothelial function and collateral development has not been evaluated in clinical studies. Endothelial function can be assessed non-invasively via flow-mediated dilation (FMD) of the brachial artery using high resolution ultrasound [12,13].

The current study was undertaken to assess whether endothelial function, measured by FMD, is associated with the extent of angiographically visible coronary collaterals in patients with high-grade coronary artery stenosis or occlusion.

## Methods

### Study population

All patients who underwent coronary angiography at our institution, and were found to have at least one major coronary occlusion, or a stenosis of ≥ 95% with Thrombolysis In Myocardial Infarction (TIMI) grade ≤ 1 anterograde flow were screened for eligibility. Patients with diagnosis of acute coronary syndrome within the past 15 days, and patients who have undergone coronary angiography using right radial approach were excluded. Patients with a history of coronary bypass surgery were also excluded if (1) the operation has been done within the past 30 days, and (2) the distal aspect of the qualifying severely stenosed or occluded artery is supplied by the patent bypass graft.

All patients gave written informed consent and local ethics committee approved the study protocol.

### Coronary angiography and collateral scoring

Coronary angiography was routinely performed by the Judkins method without the use of nitroglycerin. Percentage diameter stenosis was measured by using computerized quantitative angiography in a biplane mode (Philips DCI, Eindhoven, Netherlands). Two experienced interventional cardiologists (A.O.O and C.T.K) who were blinded to patient characteristics reviewed the angiograms and graded the coronary collaterals according to Rentrop classification [14]. In subjects with >1 collateral supplying the distal aspect of the diseased artery, the higher collateral grade was used. In subjects with >1 qualifying severely diseased vessel, the vessel with the higher collateral grade was chosen for analysis.

Intra- and inter-observer agreement of coronary collateral grades were determined from a random sample of 50 coronary angiograms (Kappa values were 0.935 for intra- and 0.845 for interobserver agreements; *p *< 0.001 for both of them). Disagreements were resolved by an additional joint reading. Patients were divided into two groups according to their collateral grades. Group 1 consisted of patients with grade 0 or grade 1 collaterals (poor collateral group) and group 2 consisted of patients with grade 2 or grade 3 collaterals (good collateral group).

### Risk factor assessment

Information about smoking, and alcohol consumption was collected through interview directly from the patients. Medical records were reviewed for reports of previous myocardial infarction, coronary artery bypass grafting, and percutaneous coronary intervention. Body mass index was calculated as weight (kg) divided by height (m^2^). Hypertension was defined as systolic blood pressure ≥ 140 mm Hg, diastolic blood pressure ≥ 90 mm Hg, or use of antihypertensive medication in the previous two weeks. Diabetes was defined as fasting blood glucose ≥ 126 mg/dL, nonfasting blood glucose ≥ 200 mg/dL, or use of antidiabetic medication in the previous two weeks. Diagnosis of acute coronary syndrome (ACS) was made according to the guidelines established by the American College of Cardiology and the American Heart Association [15]. Non-ACS patients had stable angina and/or positive stress tests.

Left ventricular ejection fraction was calculated by contrast venticulography using a standard area-length formula [16] (n = 65) or estimated by echocardiography when ventriculography was not performed (n = 106). Significant stenosis was defined as quantitatively measured diameter stenosis of >70% in any of the major coronary arteries.

### Endothelium dependent and independent flow-mediated vasodilation (FMD)

**Figure 1:**
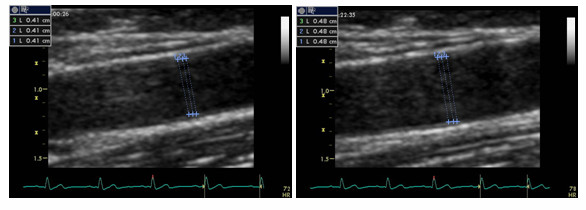
**Two-dimensional images of the brachial artery in a case with preserved endothelium dependent flow mediated dilation**. Brachial artery measurements were made from anterior intima to posterior intima at baseline, and during reactive hyperaemia. Endothelium dependent vasodilation response was 17.1%.

**Figure 2:**
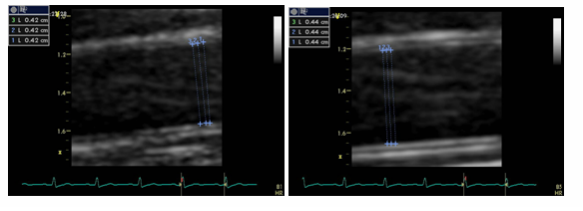
**Two-dimensional images of the brachial artery in a case with impaired endothelium dependent flow mediated dilation**. Brachial artery measurements were made from anterior intima to posterior intima at baseline, and during reactive hyperaemia. Endothelium dependent vasodilation response was 4.8%.

Endothelium dependent and independent flow-mediated dilation were performed by using a 13.0 MHz linear array transducer (Vivid 7, Wipro GE Healtcare, GE Medical Systems Inc, Chicago, U.S.A) on the day after coronary angiography. After 12 h fasting (including caffeine, nicotine and alcohol), vascular measurements were performed in a quiet, temperature controlled (22–24°C) room early in the morning (8 am-10 am). Vasoactive medications were not stopped before FMD, but the morning doses were skipped on the examination day of FMD. After 15 min resting period in the supine position, the transducer was placed 4–5 cm above the elbow in the longitudinal section for the scanning of right brachial artery, then the basal diameter of brachial artery (from anterior intima to posterior intima) and flow velocity were measured. Afterwards, sphygmomamometer cuff was placed on the upper arm, and inflated at 250 mmHg for 4–5 min, and deflated abruptly. The flow velocity was recorded within 15 seconds, and postocclusion artery diameters were taken at 60–120 seconds. After a 10 minutes resting interval, 5 mg sublingual isosorbide dinitrate was administered, and brachial artery was scanned within the next 5 minutes. Brachial artery images were taken by two experienced observers (N.U and S.T) who were blinded to the collateral status of the patients. Diameter measurements were taken at the end of the diastole (timed by peak of R wave on electrocardiogram), which were calculated at least three times, and the average of these measurements were determined. Endothelium dependent and independent vasodilations were defined as the percent change in diameter compared with baseline (maximum diameter-baseline diameter/baseline diameterx100). Two sample cases of impaired, and preserved endothelium dependent FMD measurements have been demonstrated in Figures 1 and 2. Intra- and inter-observer variability of FMD measurements were determined from 40 randomly selected brachial artery images (Kappa values were 0.93 for intra- and 0.82 for inter-observer agreements; *p *< 0.001 for both of them).

### Statistical analysis

Data are expressed as mean ± standard deviation for continuous variables, and number and percentage of subjects for categorical ones. Chi-square test with Yates correction was used to assess the significance of difference between categorical variables. When the values in the cells were not enough for chi-square test, Fischer's exact test was used. Continuous variables were compared by Student *t*-test or the Mann-Whitney rank sum test. Multivariable logistic regression analysis was used to adjust for the characteristics that were significant correlates of collateral development in the univariate analysis (with *p *< 0.1) plus terms for age and gender due to their well-established association with coronary heart disease. Odds ratios (OR) with two-tailed *p*-values and 95% confidence intervals (CI) were calculated as a measure of the association of the clinical, and therapy-related characteristics with collateral development. Statistical analyses were performed using SPSS software package (SPSS Inc., Chicago, Illinois, USA) version 16.0 for Windows and *p *< 0.05 was considered statistically significant.

## Results

A total of 257 patients met the angiographic criteria of having at least one major coronary occlusion, or a stenosis of ≥ 95% TIMI ≤ 1 anterograde flow. Eighty-six were excluded for the following reasons: (1) acute coronary syndrome within the previous 15 days (n = 53); (2) failing to give informed consent (n = 15); (3) inadequate angiograms for collateral evaluation (n = 12); (4) presence of occluded coronary artery that is supplied by the patent bypass graft (n = 6). Thus, the study group was comprised of 171 patients. Mean age of the study population was 61 years and 75% were males. Of the 171 patients, 88 (51%) had well-developed collaterals (30 patients had grade 3 and 58 patients had grade 2 collaterals) whereas 83 (49%) had impaired collateral development (37 patients had grade 1 collaterals; 46 patients had grade 0 collaterals). The prevalence of various demographic, angiographic and therapy related characteristics of the study subjects at the time of coronary angiography according to collateral classification are shown in Table 1. Subjects with poor collaterals were significantly more likely to have diabetes mellitus (*p *= 0.001) and less likely to have used statins (*p *= 0.083). The prevalence of all other cardiovascular risk factors and use of medications were similar at different levels of collaterals.

**Table 1:** Characteristics of the subjects according to collateral groups

**Variables**	**Poor collateral** **(n = 83)**	**Good collateral** **(n = 88)**	***p value***
Age (years)	61 ± 10	61 ± 11	*0.738*
Gender, males (%)	65 (78)	64 (73)	*0.396*
Diabetes mellitus (%)	46 (55)	27 (31)	*0.001*
Hypertension (%)	49 (59)	56 (64)	*0.537*
Current smokers (%)	41 (49)	39 (44)	*0.506*
Daily alcohol consumption (%)	3 (3.4)	3 (3.6)	*1.0*
BMI, kg/m^2^	28 ± 4	27 ± 4	*0.292*
Previous MI (%)	55 (66)	53 (60)	*0.413*
Previous CABG (%)	14 (17)	12 (14)	*0.556*
Total cholesterol, mg/dL	180 ± 40	179 ± 44	*0.828*
LDL-C, mg/dL	109 ± 34	107 ± 40	*0.700*
HDL-C, mg/dL	41 ± 10	39 ± 9	*0.285*
Triglycerides, mg/dL	152 ± 91	165 ± 80	*0.168*
Creatinine, mg/dL	1.1 ± 0.3	1.2 ± 0.9	*0.607*
LV ejection fraction, %	46 ± 13	49 ± 13	*0.182*
Number of vessels with significant stenosis (%)			*0.995*
1 vessel	27 (30.7)	25 (30.1)	
2 vessel	26 (29.5)	25 (30.1)	
3 vessel	35 (39.8)	33 (39.8)	
Lesion location (%)			*0.154*
Left anterior descending artery	64 (72.7)	66 (79.5)	
Circumflex artery	53 (60.2)	53 (63.9)	
Right coronary artery	67 (76.1)	55 (66.3)	
Cardiovascular medications (%)			
Aspirin	72 (87)	74 (84)	*0.623*
Beta-blockers	57 (69)	56 (64)	*0.487*
Calcium channel blockers	5 (6)	11 (13)	*0.146*
ACE-Is or ARBs	57 (69)	62 (71)	*0.800*
Nitrates	28 (34)	35 (40)	*0.413*
Statins	50 (60)	64 (73)	*0.083*

ACE-I, angiotensin converting enzyme inhibitor; ARB, angiotensin receptor blocker; BMI, body mass index; CABG, coronary artery bypass grafting; HDL-C, high density lipoprotein cholesterol; LDL-C, low density lipoprotein cholesterol; LV, left ventricular; MI, myocardial infarction

**Figure 3:**
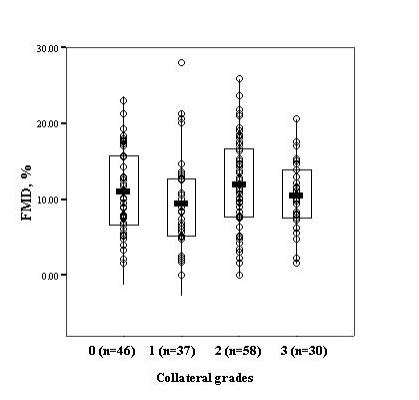
**Flow mediated dilation values according to categories of the Rentrop collateral classification**. Boxes cover the 95% confidence intervals where the line bisecting the box depicting the mean.

**Figure 4:**
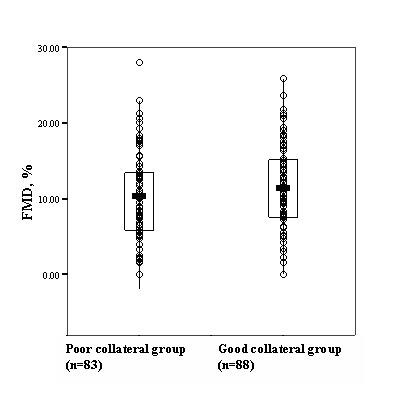
**Distribution of flow mediated dilation values by collateral groups**. Boxes cover the 95% confidence intervals where the line bisecting the box depicting the mean.

Mean FMD of the study population was 10.9 ± 5.9%, and mean nitroglycerin mediated dilation was 13.1 ± 6.2%. Figure 3 and 4 shows the mean FMD values according to categories of Rentrop collateral classification. Endothelium dependent vasodilation response was not significantly different among good and poor collateral groups (11.5 ± 5.6% vs. 10.4 ± 6.2%, *p *= 0.214). Nitroglycerin mediated dilation was also similar (13.4 ± 5.9% vs. 12.8 ± 6.5%, *p *= 0.521). We have then performed a subgroup analysis by classifying patients into 4 quartiles according to FMD results, and found that the presence of good collaterals did not differ in the first through fourth quartiles (*p *= 0.42). Also, the p value of FMD responses between quartiles 1 and 4 was still not significant [First quartile: FMD<6.6%, (n = 42), 43%; fourth quartile: FMD>14.7%, (n = 41), 57%; *p *= 0.13].

In multivariable model, diabetic patients were 2.9 times more likely to have poor collaterals than non-diabetics (95% CI: 1.6–5.7, *p *= 0.001), and patients who were not taking statins were 2 times more likely to have poor collaterals than patients taking statins (95% CI: 1.01–3.9, *p *= 0.048).

## Discussion

In this cohort of 171 patients with significant coronary artery disease we failed to find any association between FMD of the brachial artery and the extent of angiographically visible coronary collaterals. To the best of our knowledge this is the first study evaluating the relation between endothelial function and collateral formation.

Previous studies repeatedly demonstrated that nitric oxide had a pivotal role in the regulation of collateral artery development [17-19]. It has been suggested, accordingly, that endothelial dysfunction of which decreased vasodilator activity of NO is a hallmark, could be one of the potential mechanisms for the decreased presence of coronary collaterals.

One possible explanation for our negative finding is that FMD of the brachial artery examines conduit vessel response rather than the response of resistance vessels such as coronary and collateral arteries [12]. In oppose of this assumption however, many studies have demonstrated that impaired FMD is significantly associated with coronary endothelial dysfunction [20-22]. On the other hand, Anderson et al. recently showed that FMD of the brachial artery was not very sensitive (49% sensitivity) at the cut off value > 3% for the detection of coronary endothelial dysfunction and speculated FMD could not be used for the prediction of the magnitude of the coronary vasodilator response [23]. Fairly high FMD values noted in the present trial (10.9 ± 5.9%) may therefore help to explain why FMD failed to correlate with collateral score.

Another explanation for the lack of association between FMD and collateral development could be the drug effect. It has been reported that vasoactive medications have a significant effect on FMD response of brachial artery to reactive hyperemia so that it is recommended to stop these drugs at least four half-lives before FMD measurements [12,13]. Withdrawal of all vasoactive drugs in such patients with significant coronary disease, however, does not seem to be ethical. Moreover, many of these drugs were suggested to be a significant determinant of collateral formation as well [24-26] and study patients were taking them on the day of collateral evaluation. Thus we did opt to keep the medication as is. While interpreting our data however, it should be kept in mind that the responses of brachial and collateral arteries to vasoactive medications would not necessarily be the same. Indeed, vasodilator response of collateral arteries to pharmacological agents have recently been shown to be different from that of coronary arteries [27,28].

Recent data suggest that vasodilator role of NO in collateral artery development may vary depending on the duration of coronary occlusion. In an animal model of collateralogenesis it has been shown that a duration of 9 months is needed for the development of endothelium dependent relaxation mediated primarily by NO [29]. Since our data did not provide us any information about the time of coronary stenosis/occlusion, it is possible that time needed for NO to show its full vasodilatory effect has not been completed in at least some of our patients. This may have influenced our results.

It is also possible that the vasodilatory role of NO in collateral arteries might be replaced by other vasoactive influences like prostaglandins which were shown to have significant vasodilating effect on collateral vessels [30].

In conclusion, our data suggest that FMD may not be the mirror of coronary circulation and the lack of a significant relation between extent of coronary collaterals may be different from its relation of coronary artery disease extent.

## Limitations

The potential limitations of the present study are the relatively small sample size and the cross sectional study design. Despite this, all demographic and clinical variables were collected systematically using a prespecified data collection form and the prospectively collected angiographic, and laboratory data did provide us the opportunity to control for most, if not all of the potential confounders. Previous data have suggested that FMD is impaired in patients with coronary artery disease. Mean FMD values of 10.9 ± 5.9% in this cohort may, thus, be considered as unexpected. Exclusion of patients with acute coronary syndrome, high rate of statin use and performing FMD procedure without stopping the vasoactive medications may account for the fairly high FMD values observed in this trial. Nevertheless, our finding that FMD is not related with collateral formation may not be extrapolated to coronary artery disease patients with impaired FMD values. Moreover, manual ultrasonically assessment of brachial endothelial function is a noisy measurement with critical technical and interpretative limitations. It would be interesting to use a commercially available system for the automatic assessment of brachial FMD; which could be a more robust and reproducible tool.

It might be debated that Cohen-Rentrop classification is semiquantitative and might not represent the collaterals as precisely as fractional flow reserve (FFR) which is currently the gold standard for determining the presence and degree of collaterals. Although hemodynamic assessment is superior to angiographic assessment in grading collateral circulation, close correlation between Cohen-Rentrop classification and collateral FFR has been documented before [31]. Based on these data we preferred to use this widely used, practical angiographic method to grade collateral circulation.

Finally, FMD values may not reflect the true, steady state FMD values as they may be affected by the coronary angiography. However, although some reports suggested percutaneous coronary intervention procedures had an effect on FMD measurements, this was not confirmed for diagnostic coronary angiography [32].

## Conclusion

Our findings indicate that systemic endothelial function assessed via brachial artery FMD is not associated with the extent of angiographically visible coronary collateral development. Further studies are needed to evaluate the role of endothelial function on collateral formation.

## Competing interests

The authors declare that they have no competing interests.

## Authors' contributions

AOO collected data, designed and drafted the manuscript. NU, CTK, ST helped to collect data and draft the manuscript. CO, TA participated in the design of the article and performed the statistical analysis. SG, YA, CE revised the article. All authors read and approved the final manuscript.
